# No global consensus: a cross-sectional survey of maternal weight policies

**DOI:** 10.1186/1471-2393-14-167

**Published:** 2014-05-15

**Authors:** Courtney Scott, Christopher T Andersen, Natali Valdez, Francisco Mardones, Ellen A Nohr, Lucilla Poston, Katharina C Quack Loetscher, Barbara Abrams

**Affiliations:** 1 Berkeley School of Public Health, University of California, Berkeley, CA, USA; 2 Department of Anthropology, University of California, Irvine, Irvine, CA, USA; 3 Departamento de Salud Pública, Facultad de Medicina, Pontificia Universidad Católica de Chile, Santiago, Chile; 4 Department of Gynecology and Obstetrics, Odense University Hospital, Odense, Denmark; 5 Department of Public Health, Aarhus University, Aarhus, Denmark; 6 Division of Women’s Health, King’s College London, St Thomas’ Hospital, London, UK; 7 Department of Obstetrics, University Hospital Zurich, Zurich, Switzerland; 8 103 Haviland Hall, Berkeley, CA 94720, USA

**Keywords:** Maternal weight policies, Key informant, International

## Abstract

**Background:**

Growing evidence suggests that maternal prepregnancy weight and gestational weight gain are risk factors for perinatal complications and subsequent maternal and child health. Postpartum weight retention is also associated with adverse birth outcomes and maternal obesity. Clinical guidelines addressing healthy weight before, during, and after pregnancy have been introduced in some countries, but at present a systematic accounting for these policies has not been conducted. The objective of the present study was to conduct a cross-national comparison of maternal weight guidelines.

**Methods:**

This cross sectional survey administered a questionnaire online to key informants with expertise on the subject of maternal weight to assess the presence and content of preconceptional, pregnancy and postpartum maternal weight guidelines, their rationale and availability. We searched 195 countries, identified potential informants in 80 and received surveys representing 66 countries. We estimated the proportion of countries with guidelines by region, income, and formal or informal policy, and described and compared guideline content, including a rubric to assess presence or absence of 4 guidelines: encourage healthy preconceptional weight, antenatal weighing, encourage appropriate gestational gain, and encourage attainment of healthy postpartum weight.

**Results:**

Fifty-three countries reported either a formal or informal policy regarding maternal weight. The majority of these policies included guidelines to assess maternal weight at the first prenatal visit (90%), to monitor gestational weight gain during pregnancy (81%), and to provide recommendations to women about healthy gestational weight gain (62%). Guidelines related to preconceptional (42%) and postpartum (13%) weight were less common. Only 8% of countries reported policies that included all 4 fundamental guidelines. Guideline content and rationale varied considerably between countries, and respondents perceived that within their country, policies were not widely known.

**Conclusions:**

These results suggest that maternal weight is a concern throughout the world. However, we found a lack of international consensus on the content of guidelines. Further research is needed to understand which recommendations or interventions work best with respect to maternal weight in different country settings, and how pregnancy weight policies impact clinical practices and health outcomes for the mother and child.

## Background

There is growing evidence that pregnancy is a critical time to establish lifelong health for the mother and her offspring, with potential to interrupt the growing worldwide epidemics of obesity and non-communicable disease [1-4]. Pre-pregnancy obesity and excessive gestational weight gain increase perinatal complications, contribute to the transmission of obesity and poor health to the next generation and increase permanent adiposity and related disease outcomes in the mother [5-9]. Interpregnancy weight gain, which may reflect postpartum retention of gestational weight gain or new weight gain between pregnancies, has been associated with serious adverse pregnancy outcomes, even among mothers whose pre-pregnancy body mass index (BMI) is in the normal range for both first and second pregnancies [10]. Fetal growth restriction resulting from maternal pre-pregnancy underweight and inadequate gestational weight gain [11,12] may also contribute to poor adult health, as low birthweight, particularly when accompanied by catch-up growth, is associated with less lean body mass, higher visceral fat and increased metabolic syndrome in adulthood [13]. Overall, the current evidence suggests that beginning pregnancy at a healthy weight (BMI 18.5-24.9 kg/m^2^), having an appropriate amount of gestational weight gain (GWG) and losing excess weight postpartum are beneficial for both the short and long-term health of a mother and her child [14-19].

In 2009, the United States Institute of Medicine (IOM) issued evidence-based clinical guidelines designed to improve maternal and child health by recommending a healthy maternal BMI before pregnancy, assessing pre-pregnancy BMI at the first prenatal visit, providing women with a recommended gestational weight goal based on her pre-pregnancy BMI, counseling on healthy lifestyle, and monitoring weight gain throughout pregnancy [16,20]. The United Kingdom’s National Institute for Health and Clinical Excellence (NICE) also recommends beginning pregnancy at a healthy BMI, counselling on healthy lifestyle during pregnancy and encouraging return to a healthy BMI after birth. However, NICE concludes there is insufficient experimental evidence that addressing gestational weight gain will lead to improved birth outcomes. NICE therefore recommend against routine maternal weighing in pregnancy and does not recommend a GWG guideline [21].

While these pregnancy weight guidelines have been developed in high income countries such as the United States and United Kingdom, where obesity and excessive gestational weight gain are common, guidelines for maternal weight management in other countries around the world may also be needed. More than 40% of women of childbearing age in Africa and 70% in the Americas and the Caribbean are now overweight or obese, while in Asia and Africa maternal underweight remains a major concern, with a prevalence of more than 10% [22]. In Japan, low maternal pre-pregnancy BMI and low gestational weight gain have contributed to a decrease in term birth weight observed over the past two decades [23]. The differences between the UK and the US policies and a 2013 review that compared national GWG guidelines and dietary energy recommendations [24] suggest that there may be important differences to explore between national policies related to maternal weight. Here we report an online cross-national key informant survey that sought to systematically compare national policies and guidelines related to maternal weight before, during and after pregnancy.

## Methods

### Online search

In 2010, we conducted a search of PubMed and Internet search engines, using the key words ‘maternal weight policy’ and ‘pregnancy weight policy’ in English, French, German and Spanish to identify written documents describing national guidelines addressing maternal weight before, during and after pregnancy. Finding data on only a few countries, we designed an online key informant [25] survey to investigate formal and informal national policies and guidelines related to maternal weight status before, during, and after pregnancy. For this study, we define a “policy” as a broad set of guidelines or recommendations incorporating multiple aspects of pregnancy weight and nutrition. We will refer to the specific components of the policies as “recommendations” or “guidelines”. We defined ‘formal’ policies as those that have been adopted or endorsed by a government body (e.g. Ministry of Health) or a professional organization (e.g. College of Obstetricians and Gynaecologists) and “informal” as those policies clinicians follow without such endorsement.

### Key informants

Our sampling frame was the list of 195 independent states recognized by the U.S. Department of State in 2011 [26], as well as Hong Kong and Scotland, territories that have independent policies related to maternal weight. As we were unable to identify a global database of maternal weight or nutrition experts from which to sample, and since the total population of individuals who would fit into this category was assumed to be relatively small, we used a convenience sample. We sought one respondent per country, and defined an eligible key informant as a government health official, leader of a professional organization, clinician, academic or researcher with expertise in perinatal health and national nutrition policy. We initially identified potential key informants through professional and research networks and through PubMed and Google Scholar. The search terms ‘gestational weight’ , ‘maternal weight’ , or ‘pregnancy weight’ were used in combination with the name of the country where an informant was sought. Literature searches were conducted in English, French, German and Spanish.

**Figure 1:**
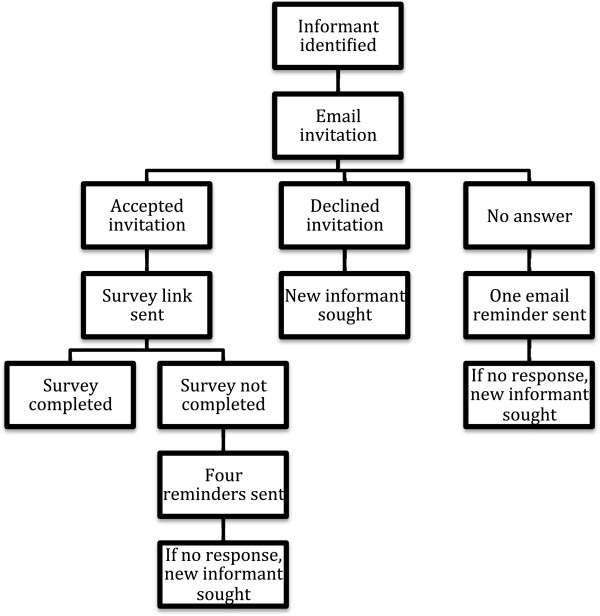
Data collection protocol.

A standard invitation was sent by email to each potential key informant describing the study, assessing whether the person had expertise about maternal weight policies and recommendations for their country and was willing to respond to the survey. If a potential informant was unwilling to participate, we requested that the informant recommend another key informant in their country. If we did not receive a response from a potential informant after two email attempts, we continued to search for additional informants until we enrolled a key informant from as many countries as possible. Four reminders were sent to informants who received but did not complete the survey before we sought out an additional informant (Figure 1).

### Survey

The survey consisted of 29 closed-ended questions with optional qualitative comment boxes, and was designed for administration on the Internet [27] using Qualtrics survey software [28]. The content of the survey was based on concepts and terms from the 2009 US IOM *Weight Gain During Pregnancy* Guidelines [20], the 2010 UK *Weight Management Before, During and After Pregnancy* Guidelines [21], and a previous survey on weight gain in pregnancy among midwives in the UK [29]. The study investigators, who represent five countries (Chile, Denmark, Switzerland, the UK, and the US), collectively drafted the questionnaire to ensure that important domains were included and questions were clear. The survey was pre-tested on 17 physicians, midwives, researchers and policy experts from three countries (the US, the UK and Switzerland), and their feedback was incorporated in a revised questionnaire. The survey was also translated into Spanish and tested by two native Spanish speakers.

The survey quantitatively assessed the presence and content of guidelines related to pre-pregnancy weight, routine weighing during pregnancy, gestational weight gain guidelines and postpartum weight. Questions also assessed the scientific basis or rationale for select guidelines. Key informants were asked to estimate the extent to which the policies were clear, widely known and available to health professionals. We requested a copy or citation for the written policy document, if available. The survey also provided an opportunity for respondents to provide comments, which are not reported here. Table 1 summarizes the main questions from the survey (full survey instrument is available upon request).

**Table 1:** Main survey questions

**Question**	**Sub-question**
Does your country have a formal policy related to maternal weight?	If not, does your country have an informal policy related to maternal weight?
Does the policy recommend beginning pregnancy at a healthy weight and to provide pre-pregnancy nutrition counseling?	If so, for which women?
Does the policy recommend assessing pre-pregnancy weight during prenatal care?	If so, how is it assessed?
Does the policy specify to weigh women during pregnancy?	If so, how often and for which women?
Does the policy specify a recommended amount of gestational weight to gain?	If so, how is the GWG recommendation determined? Is it determined by BMI?
Does the policy recommend assessing post-partum weight status?	
Does the policy specify to provide post-partum counseling on weight or nutrition?	If so, for which women?

### Protocol

We administered the survey from January 2012 to May 2013. Although only one key informant per country was sought, we received simultaneous affirmation from two informants for Brazil, China, South Africa, and Ireland, and the survey was administered to both informants. In the three cases where the responses from the two informants in the same country did not agree, we sought clarification from the informants. If the original policy document was provided to us, the informant whose answers matched that of the policy document was selected for inclusion in the study.

### Analysis

We assessed the percentage of countries with guidelines, and grouped countries by World Health Organization (WHO) region [30], World Bank income group [31], and whether the respondent indicated their country had a formal or informal policy, using Qualtrics and Stata software [32]. To standardize comparison of policy content reflecting maternal pre-pregnancy, pregnancy and postpartum weight status across the countries surveyed, we created a rubric to assess the presence or absence of four guidelines we considered fundamental to perinatal health and/or are a topic of debate in the field: begin at a healthy pre-pregnancy weight, routinely weigh at antenatal visits, provide gestational weight gain guidelines, and promote healthy post-partum weight. We then calculated the proportion of countries with guidelines addressing each of these topics and classified each country according to the number reported, with results ranging from four to zero. It is important to note that a country with a ‘zero’ score could have a maternal weight or nutrition guideline other than the four considered in our rubric.

### Ethical approval

Staff at the Office of Protection of Human Subjects at the University of California, Berkeley reviewed the survey and protocol. They determined that this study did not require approval or exemption according to their guidelines for human subjects research, as it did not focus on characteristics of an individual or groups of individuals and the information collected about national policies is not about the informants themselves.

## Results

We searched 195 countries, identified potential key informants for 80 countries and collected 66 surveys (64 countries plus Hong Kong and Scotland). Table 2 displays the responding countries by WHO region, income level and reported policy status. Though we had representation from all regions of the world, the majority of the surveys represented high and middle-income countries, mostly in Europe and the Americas. African (n = 5) and South-East Asian (n = 4) countries were less represented. The majority of key informants identified themselves as researchers or clinicians (Table 3).

**Table 2:** Respondent countries by WHO region and maternal weight policy

	***Africa***	***Americas***		***Eastern Mediterranean***	***Europe***		***South-East Asia***	***Western Pacific***
	***(8%, n = 5)***	***(27%, n = 18)***		***(12%, n = 8)***	***(36%, n = 24)***		***(6%, n = 4)***	***(11%, n = 7)***
*Formal maternal weight policy*	South Africa^†^	Argentina^†^	Ecuador^†^	Iran^†^	Belgium*	Netherlands*	India^‡^	Australia*
		Bolivia^‡^	Guatemala^‡^		Bulgaria^†^	Norway*	Myanmar^§^	China^†^
		Brazil^†^	Honduras^‡^		Croatia*	Poland*		Japan*
		Canada*	Nicaragua^‡^		Denmark*	Portugal*		Vietnam^‡^
		Chile^†^	Paraguay^‡^		Finland*	Romania^†^		
		Costa Rica^†^	Peru^†^		France*	Russia^†^		
		Cuba^†^	United States of America*		Ireland*	Sweden*		
			Uruguay^†^		Italy*	Switzerland*		
						United Kingdom*		
*Informal maternal weight policy*	Ghana^‡^	Mexico^†^		Oman*	Germany*	Scotland*	Bangladesh^§^	New Zealand*
	Tanzania^§^	Venezuela^†^		Pakistan^‡^	Lithuania^†^			
	Zambia^‡^			United Arab Emirates*				
*No maternal weight policy*	Nigeria^‡^	Colombia^†^		Egypt^‡^	Iceland*		Thailand^†^	Hong Kong*
				Lebanon^†^	Macedonia^†^			Singapore*
				Saudi Arabia*	Israel*			
				Sudan^‡^	Spain*			

*High income, † Upper-middle income, ‡ Lower-middle income, § Low income.

**Table 3:** Characteristics of sampled countries

		
**Income group**	**n = 66**	**%**
High	29	43.9
Upper-middle	21	31.8
Lower-middle	13	19.7
Low	3	4.5
**Maternal weight policies**	**n = 66**	**%**
Formal	40	60.6
Informal	13	19.7
None	13	19.7
Respondent profession*	**n = 66**	**%**
Researcher	42	63.6
Clinician	30	45.5
Nutritionist	10	15.2
Other	8	12.1
Government	6	9.1
**Issuer of formal policy**	**n = 40**	**%**
Government	22	55
Professional organization	8	20
Partnership or other	10	25

*Respondents were allowed to indicate more than one profession.

Table 3 shows that 40 countries reported having a formal maternal weight policy, 13 countries reported an informal policy and 13 had no policy. The study results are based on the 53 countries that reported a policy. About half of the formal policies were government issued.

### Maternal weight status

Forty two per cent of country policies (n = 22) included a guideline to *begin pregnancy at a healthy weight*. All but five of the 53 national policies (90%) recommended assessing *weight at the first prenatal visit*, with 46 measuring weight at that time and 15 using self-reported pre-pregnancy weight. Forty- three countries (81%) reported the practice of *routine weighing* all mothers at each antenatal visit. Three reported weighing only women who were underweight or overweight/obese at the first visit, and 7 countries weigh “only if clinical management can be influenced or nutrition is a concern”. Only 13% (n = 7) of country policies included a guideline to support all women returning to a *healthy postpartum weight*, although four additional countries had a postpartum policy for some women*.* See Additional file 1 for details on which policies were reported for each country.

### Gestational weight gain guidelines

Sixty two per cent of countries included *guidelines for recommended GWG in their policy* (Table 4). Nineteen countries recommended ranges by pre-pregnancy BMI category. Among these, thirteen countries reported GWG BMI guidelines similar to the US IOM guidelines (within 1 kg on either side of the IOM recommendation) in at least two BMI weight categories. Six countries reported lower ranges than the IOM guidelines or a single value for each BMI category. Recommendations were more variable for women with pre-pregnancy BMI greater than 30. Eight countries reported guidelines for obese women that were identical to or similar to the 5–9 kg recommended by the US IOM, but other countries recommended different amounts, as ranges, single values or representing upper or lower limits. Japan was unique in recommending an individualized approach for all women with a pre-pregnancy BMI greater than 25. Eight countries from Latin America did not use a pre-pregnancy BMI category, but rather based their recommendations on achieving a target BMI at a given gestational age, such as the Rosso-Mardones Chart [33]. Six countries reported a variety of approaches not linked to the pre-pregnancy BMI or gestational age, including a single recommended value, a single range, or a recommended monthly gain.

**Table 4:** Gestational weight gain recommendations by country

**Country**	Recommendations by pre-pregnancy BMI category*					
	**<18.5 kg/m^2**	**18.5-24.9 kg/m^2**	**25-29.9 kg/m^2**	**30-34.9 kg/m^2**	**35-39.9 kg/m^2**	**>40 kg/m^2**
United States	**12.5 - 18**	**11.5 - 16**	**7 - 11.5**	**5 - 9**	**5 - 9**	**5 - 9**
Bulgaria	*12 - 18*	*11 - 16*	*7 - 11*	**5 - 9**	*5 - 8*	*5 - 8*
Ghana	**12.5 - 18**	**11.5 - 16**	**7 - 11.5**	*5 - 10*	*5 - 10*	*5 - 10*
Italy	**12.5 - 18**	**11.5 - 16**	**7 - 11.5**	**5 - 9**	**5 - 9**	**5 - 9**
Canada	**12.5 - 18**	**11.5 - 16**	**7 - 11.5**	**5 - 9**	**5 - 9**	**5- 9**
Nicaragua	*12.7 - 18.1*	*11.3 - 15.9*	*6.8 - 11.3*	*5 - 9.1*	None given	None given
Denmark	*13 - 18*	10 - 15	8 - 10	*6 - 9*	*6 - 9*	*6 - 9*
Poland	**12.5 - 18**	*11.4 - 15.9*	*6.5 - 11.4*	7 (upper limit)	7 (upper limit)	7 (upper limit)
Romania	**12.5 - 18**	**11.5 - 16**	**7 - 11.5**	7 - 11.5	7 - 11.5	7 - 11.5
Switzerland	**12.5 - 18**	**11.5 - 16**	**7 - 11.5**	7 (upper limit)	7 (upper limit)	7 (upper limit)
Brazil	**12.5 - 18**	**11.5 - 16**	**7 - 11.5**	7 (no range)	7 (no range)	7 (no range)
Paraguay	**12.5 - 18**	11.5 - 14	**7 - 11.5**	*6 - 8*	None given	None given
Iran	12 - 18	9 - 14	**7 - 11.5**	6 (no range)	6 (no range)	6 (no range)
China	14 - 15	12 (no range)	7 - 8	7 - 8	7 - 8	7 - 8
Croatia	14 (upper limit)	12 (upper limit)	10 (upper limit)	8 (lower limit)	6 (lower limit)	4 (lower limit)
Cuba	9.45 - 17	8.6 - 15.9	7.5 - 14	5.4 - 12.9	5.4 - 12.9	5.4 - 12.9
Japan	9 - 12	7 - 12	individual	individual	individual	individual
Portugal	6 - 12	5 - 10	5 - 7	5 - 7	5 - 7	5 (no range)
Russia	12 (no range)	12 (no range)	10 (no range)	10 (no range)	8 (no range)	8 (no range)
	**Recommendations by BMI at a specific gestational age chart**					
Argentina	Country-specific guideline chart					
Bolivia	Rosso and Mardones^†^					
Chile	Atalah, et al.^§^					
Ecuador	Rosso and Mardones^†^					
Guatemala	Atalah, et al.^§^					
Honduras	Country-specific guideline chart					
Peru	Rosso and Mardones^†^					
Uruguay	Atalah, et al.^§^					
	**Other recommendations not based on body size**					
Burma	1 kg per month from month 5 of gestation to term					
France	Average gain around 12 kg					
India	10 - 12 kg					
Oman	Client materials recommend gaining 9–15 kg					
Vietnam	9 - 12 kg					
South Africa	Formal recommendation that women should not be given a guideline for weight gain in pregnancy					

*Bolded text indicates that a weight gain recommendation exactly matches the IOM recommendations. *Italicized text indicates that a weight gain recommendation falls within 1 kg of either side of the U.S. IOM recommendations*.

†Mardones F, Rosso P. A weight gain chart for pregnant women designed in Chile. Matern Child Nutr. 2005.

§Atalah E, Castillo C, Castro R, Aldea A. Proposal of a new standard for the nutritional assessment of pregnant women. Rev Med Chil. 1997.

### Reasons for policies

The questionnaire asked key informants to select from a list of reasons or evidence underlying the reported guidelines for routine weighing and recommended gestational weight gain. Table 5 shows that reported reasons for routine weighing during pregnancy included monitoring weight gain so as to promote a healthy GWG and to assess maternal or fetal health. The 7 countries without a guideline to routinely weigh pregnant women cited lack of evidence that weighing improves health (6 countries), a lack of time or equipment (3 countries), and concerns about maternal anxiety (2 countries). Countries with a GWG recommendation indicated that the guidelines were based on attempts to reduce adverse maternal and infant health outcomes. Only one country reported explicit recommendations against specifying a GWG amount, citing lack of research informing the ideal amount and lack of evidence for improved birth outcomes.

**Table 5:** Reported basis for pregnancy weight guidelines

	**Is routine weighing recommended?**	**Are there gestational weight gain guidelines?**
	**Yes (n = 43)**	**Yes (n = 32)**
	***n (%)***	***n (%)***
Promote healthy gestational weight gain	41 (95%)	N/A
Avoid maternal complications	29 (67%)	26 (81%)
Avoid poor birth outcomes	25 (58%)	25 (78%)
Avoid long term maternal obesity	N/A	17 (53%)
Other	4	3 (9%)

### Counselling guidelines

Table 6 displays the prevalence of reported counselling before and during pregnancy for weight and lifestyle behaviours. Approximately two thirds of countries recommended providing counselling on healthy weight before and during pregnancy. Almost three quarters recommended supplemental folic acid and healthy diet before pregnancy. Overall, counselling about healthy diet and physical activity was more common preconceptionally than during pregnancy, and physical activity was less discussed than weight or diet.

**Table 6:** National maternal policies that include guidelines for counselling before and during pregnancy

	**Preconception (n = 22)**	**During pregnancy (n = 53)**
	***n (%)***	***n (%)***
Promote healthy weight	15 (68%)	35 (66%)
Physical activity	14 (64%)	24 (45%)
Healthy diet	16 (73%)	34 (64%)
Folic acid supplements	16 (73%)	N/A
Other	N/A	10 (19%)

### Perceptions of guidelines

Key informants reported their perceptions of the accessibility of their national maternal weight policies (Table 7). While 85% felt that guidelines were clear for the pregnancy period, half or less reported clarity for pre or post pregnancy guidelines. About two thirds reported that guidelines were easy to obtain for the pregnancy and postpartum period, but this proportion fell to below half for the prepregnancy period. The proportion of respondents who reported that guidelines were widely known differed for the preconceptional (27%), pregnancy (52%) and postpartum (37%) periods.

**Table 7:** Key informant perceptions of maternal weight policies

	**Prepregnancy weight (n = 22)**	**Gestational weight gain (n = 33)**	**Postpartum weight (n = 11)**
	***n (%)***	***n (%)***	***n (%)***
Guidelines are clear	11 (50%)	28 (85%)	5 (45%)
Guidelines are easy to obtain	10 (45%)	22 (67%)	7 (64%)
Guidelines are widely known by health professionals	6 (27%)	17 (52%)	4 (37%)

### Guideline coverage across the childbearing period

To compare the content of the 53 policies we used the rubric described in the methods section that contained four fundamental guidelines addressing maternal weight before, during and after pregnancy: beginning pregnancy at a healthy weight, providing a target GWG, monitoring GWG, and returning to a healthy postpartum weight. Eight per cent of the national policies included all four guidelines (Additional file 1). Zero, one, two and three guidelines were included in 8%, 25%, 38% and 23% of national policies, respectively. Countries with formal policies were more likely to cover all four areas than those with informal policies.

### Written policies

Twenty-three countries provided written policies.

## Discussion

Key informants in 66 countries completed the survey, and 53 informants reporting a policy, of which 40 were reported as formal and 13 informal. The majority of the policies addressed the pregnancy period, with guidelines to assess maternal body weight at first visit (90%), to monitor GWG throughout pregnancy (81%) and to have a policy addressing GWG (62%). However, the content of these prenatal guidelines varied across nations. Less than 10% of national policies addressed healthy maternal weight across the entire spectrum of childbearing, from preconception through postpartum. Less than half of the policies included guidelines to promote healthy maternal weight before pregnancy, and only 13% of the policies addressed healthy weight during the postpartum period. The content of GWG guidelines differed across the world. More than half of the countries anchored their GWG target on pre-pregnancy BMI, but the recommended GWG amounts were inconsistent, particularly for obese women. Some GWG guidelines were clearly modelled on the US IOM guidelines, while other countries with BMI-specific goals were not. Even when guidelines existed, respondents indicated that dissemination was limited. These results suggest that maternal weight is a concern throughout the world, but that there is a lack of international consensus on the content of guidelines.

The majority of the key informants reported a guideline in their country to routinely weigh during pregnancy. Respondents from countries that monitored weight indicated that the practice is aimed at reducing adverse perinatal outcomes, while those from countries who did not recommend routine weighing reported concerns that weighing may not impact outcomes and may also increase maternal anxiety. There is little question from observational studies that extremes of total or early GWG are associated with poor health outcomes [20], but there is very little evidence to support or refute the effectiveness of weighing alone to improve either GWG or maternal and child health. To our knowledge, the single randomized trial evaluating routine weighing (combined with a GWG goal but without further counselling) reported no significant effect on total GWG [34]. The lack of efficacy of measuring weight alone is not surprising, given that systematic reviews of recent randomized controlled trials suggest that decreasing excessive GWG is challenging, and may require a package combining routine weighing and effective lifestyle interventions aimed at optimizing maternal weight and metabolic status as well as addressing psychological factors and barriers in the greater social environment [35-37]. However, there are examples of comprehensive interventions that effectively reduced excessive GWG in some groups (e.g. normal weight women) [38] and increased GWG and resultant birth weight in an undernourished population [39]. In the present survey, more than 80% of national policies included a guideline to routinely monitor GWG, but only 60% also included a GWG guideline to use in the assessment and counselling that studies suggest may be important for effective intervention. Furthermore, although some countries cite maternal anxiety as a reason to not routinely weigh, we were unable to find empirical evidence supporting this. New data are needed to establish the effectiveness of measuring weight routinely in pregnancy, alone or within a package of services, as well as possible psychological harms that could be associated with repeatedly weighing.

Policies for preconceptional weight were reported in 22 countries (42%), with 15 explicitly recommending achieving a healthy weight before conceiving. Only seven national policies (13%) reported guidelines for measuring, assessing or counselling on post-partum weight. The absence of postpartum weight policies is particularly concerning as evidence shows that weight gain in pregnancy may be retained after the birth and carry into subsequent pregnancies [10,18] – potentially “propagating the cycle of obesity” described by Catalano [4]. Only 4 countries reported a policy with guidelines aimed at promoting healthy weight before, during and after pregnancy.

Our study had several limitations. While we sought a key informant in every country in the world using published research, Internet resources and professional networks, we were unable to identify key informants in 112 countries. However, we received responses in 80% of the countries where we did identify a key informant. The majority of our respondents came from the Americas and Europe, as we were unable to identify many key informants from African countries and South-East Asia, although we did have representation from every WHO region. Our search for key informants was limited to only four languages. Our ability to identify key informants was also limited by the fact that the survey was administered only in English and Spanish, and also required access to the Internet. The majority of policies described in this study came from high or upper middle-income countries, thus our findings are not as applicable to less developed nations.

With no obvious sampling frame to identify experts in maternal nutrition, we utilized a single key informant to report on each country’s policy and guidelines, and thus the validity and reliability of our results are entirely dependent upon the knowledge and expertise of that informant. In three of the four countries where we had two informants, the informants did not agree on all responses, which raise questions about the validity of the key informant approach. Two cases of disagreement were over whether there was a policy for that country, and one was whether the policy was formal or informal in nature. In all of these cases, one informant provided us with a copy of the policy for their country, so we reported that informant’s responses. These disagreements are additional evidence that policies and guidelines related to maternal weight are not clear or well known even among those who identify themselves as experts. We cannot assess how widespread the disagreement between key informants would have been if we had surveyed multiple key informants in every country, but we believe our findings are an important first step towards understanding the global policy landscape. Future research is needed to confirm and expand upon our findings.

The key informant approach was also a unique strength of this study. To the best of our knowledge, only one other published paper has assessed national maternal weight policies [24]. Alavi and colleagues performed an Internet search of pregnancy weight and recommended energy intake policies and identified 14 GWG policies. Our approach yielded twice as many policies, in part because we queried both formal and informal policies, and we were able to identify policies in languages other than English or not published on the Internet. Furthermore, our study examined policies for routine weighing and pre- and post- pregnancy weight recommendations.

The variety of policies and recommendations found in our study leaves many questions for further exploration. Does having a maternal weight policy influence clinical outcomes, and if so what specific recommendations are most influential? Is there a benefit to having a national maternal weight policy? How do nations develop or select their maternal weight policies and recommendations? Is there justification or adequate evidence for standardization of maternal weight policies across countries, or do policies need to be tailored for specific contexts? Is a lack of adequate evidence, particularly related to each individual country, the reason for the varied guidelines? Are there country-level patterns related to maternal weight that would benefit from cross-cultural examinations to challenge the assumptions made? Further research is needed to answer these questions, ranging from country-level case studies exploring the relationship between policies/guidelines and clinical outcomes to large, perhaps even multinational, trials looking into the effectiveness of specific guidelines or practices related to maternal weight. We believe the World Health Organizations, United Nations Children’s Fund, or other United Nations organizations are particularly well suited to conduct such work.

## Conclusion

Despite the potential impact of healthy maternal weight on maternal and child health, our survey results indicate inconsistencies across countries in national policies and recommendations throughout the world, echoing and expanding results of a previous study [24]. Our study contributes new findings that document how practices related to establishing a healthy weight before and after pregnancy, assessing pre-pregnancy or early pregnancy weight, monitoring weight change during pregnancy and providing lifestyle counselling to improve maternal health vary widely. Even within countries, respondents reported limited dissemination and awareness of formal maternal policies. Results from trials aimed at determining the most effective methods for improving maternal weight, nutrition, metabolic health and clinical outcomes are needed. In addition, our results suggest that a systematic, formal worldwide review of pregnancy weight policies, including their impact on clinical practices and health outcomes for the mother and child, would be a valuable step toward improving the health of mothers and children throughout the globe.

## Abbreviations

BMI: Body mass index; GWG: Gestational weight gain; IOM: Institute of medicine; NICE: National Institute for Health and Clinical Excellence; UK: United Kingdom; US: United States; WHO: World Health Organization.

## Competing interests

The authors declare no personal or financial competing interests.

## Authors’ contributions

CS, BA, LP, FM, EN, and KQL formulated the research question and designed the study. CS, BA, and CA conducted data collection, analyzed the data, and wrote the manuscript. LP, FM, EN, and KQL contributed to analysis and manuscript preparation. All authors have read and approved the final manuscript.

## Pre-publication history

The pre-publication history for this paper can be accessed here:

http://www.biomedcentral.com/1471-2393/14/167/prepub

## Supplementary Material

Additional file 1 **Four key policies by country.** This rubric compares the content of 53 policies, and assesses the presence of the following four components: beginning pregnancy at a healthy weight, providing a target GWG, monitoring GWG, and returning to a healthy postpartum weight. Click here for file
