# Supplementary material for: No global consensus: a cross-sectional survey of maternal weight policies
Source: BMC Pregnancy Childbirth. 2014 May 15;14:167. doi: 10.1186/1471-2393-14-167 (PMC4031379; doi:10.1186/1471-2393-14-167)
Supplement: Additional file 1 — Four key policies by country. This rubric compares the content of 53 policies, and assesses the presence of the following four components: beginning pregnancy at a healthy weight, providing a target GWG, monitoring GWG, and returning to a healthy postpartum weight. [file 1471-2393-14-167-S1.pdf]

Additional File 1: Four key policies by country

Country	Type of policy	Guideline topic area				Total policies
		Pre-pregnancy weight	Routine weighing in pregnancy	Gestational weight gain	Post-partum weight	
**Argentina**	Formal		X	X		2
**Australia**	Formal					0
**Belgium**	Formal	X	X			2
**Bolivia**	Formal		X	X		2
**Brazil**	Formal		X	X		2
**Bulgaria**	Formal	X	X	X		3
**Burma**	Formal		X	X		2
**Canada**	Formal	X	X	X		3
**Chile**	Formal	X	X	X	X	4
**China**	Formal		X	X	X	3
**Costa Rica**	Formal		X			1
**Croatia**	Formal		X	X		2
**Cuba**	Formal	X	X	X	X	4
**Denmark**	Formal		X	X		2
**Ecuador**	Formal		X	X		2
**Finland**	Formal		X			1
**France**	Formal		X	X		2
**Guatemala**	Formal		X	X		2
**Honduras**	Formal	X	X	X		3
**India**	Formal	X	X	X		3
**Iran**	Formal		X	X		2
**Ireland**	Formal	X				1
**Italy**	Formal	X	X	X	X	4
**Japan**	Formal	X	X	X		3
**Netherlands**	Formal	X				1
**Nicaragua**	Formal	X	X	X		3
**Norway**	Formal		X			1
**Paraguay**	Formal		X	X		2
**Peru**	Formal		X	X		2
**Poland**	Formal	X	X	X		3
**Portugal**	Formal	X	X	X		3
**Romania**	Formal	X	X	X	X	4
**Russian Federation**	Formal		X	X		2
**South Africa**	Formal			X		1
**Sweden**	Formal		X		X	2
**Switzerland**	Formal		X	X		2
**United Kingdom**	Formal	X			X	2
**United States of America**	Formal	X	X	X		3

**Uruguay**	Formal	X	X	X		3
**Vietnam**	Formal	X	X	X		3
**Bangladesh**	Informal		X			1
**Germany**	Informal					0
**Ghana**	Informal		X	X		2
**Lithuania**	Informal		X			1
**Mexico**	Informal	X	X			2
**New Zealand**	Informal					0
**Oman**	Informal			X		1
**Pakistan**	Informal		X			1
**Scotland**	Informal	X				1
**Tanzania**	Informal		X			1
**United Arab Emirates**	Informal	X	X			2
**Venezuela**	Informal					0
**Zambia**	Informal		X			1
